# Severe lupus after two years of hemodialysis: It exists and can be serious

**DOI:** 10.1186/s43162-023-00208-1

**Published:** 2023-03-30

**Authors:** Marouane Jabrane, Mohammed Bouchoual, Mohamed Arrayhani

**Affiliations:** Department of Nephrology and Kidney Transplantation, Hassan II Hospital, Faculty of Medicine and Pharmacy, Medical University Hospital, Ibn Zohr University, Agadir, Morocco

**Keywords:** Lupus flare, Hemodialysis, Spontaneous ecchymosis

## Abstract

Progression of lupus nephropathy (LN) to end-stage renal disease is a serious complication and requires subsequent replacement therapy. Lupus disease activity is extinguished in chronic hemodialysis. We report the observation of a 35-year-old female patient, in conventionnel hemodialysis for two years (chronic glomerulonephritis), admitted to the emergency room for convulsions, left flaccid tenderness, cutaneous-mucosal pallor and altered general condition evolving since three days before her admission. we also observed a spontaneous ecchymotic lesions on the right arm. Echodoppler of the right upper extremity was in favor of a partially thrombosed aneurysm of the right brachial artery. The biological workup showed pancytopenia, the requested immunological workup showed a low complement C_3_, a positive level of anti-DNA antibodies. The patient was treated as severe lupus flare: Bolus of methylprednisolone, followed by oral administration, associated with Mycophenolate mofétil (MMF) at a dose of 1 g/d. The evolution was favorable on the clinical, biological and radiological levels.

Systemic lupus erythematous (SLE) can occur even after several years of hemodialysis and sometimes in a severe form, pushing the clinician to think of this pathology in the presence of evocative signs.

## Case report

Young woman, 35 years old, in conventionnel hemodialysis for two years due possibly to chronic glomerulonephritis with a low positivity of anti DNA antibodies without further etiological research. She is admitted to the emergency room for tonic–clonic, generalized convulsions, of abrupt onset without any other associated signs.

**Fig. 1:**
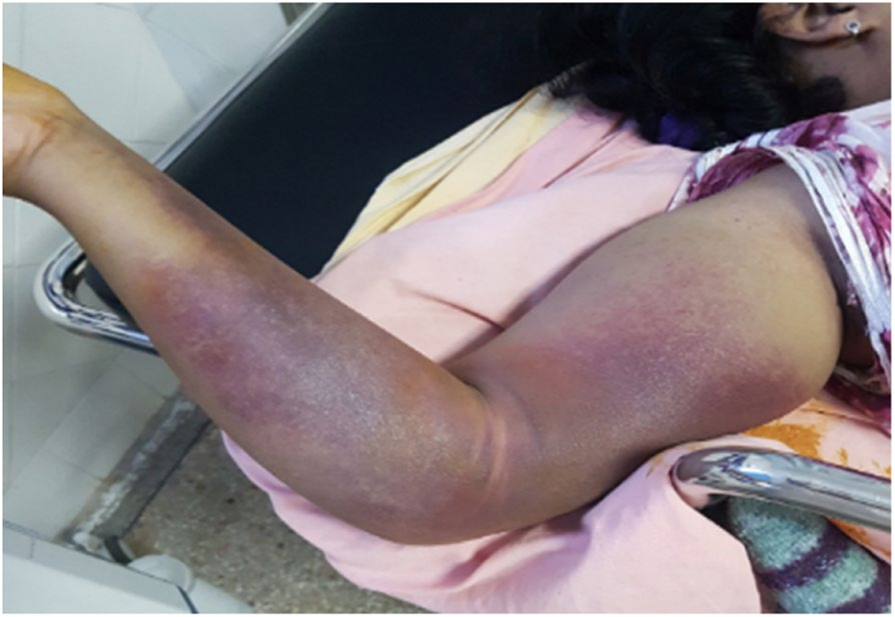
Spontaneous ecchymosis of the right arm with thrombosed aneurysm of the right brachial artery

Clinical examination on admission revealed a confused patient, febrile at 38.6 °C, hemodynamically and respiratory stable with generalized mucocutaneous pallor. A spontaneous ecchymosis was noted on the limb contralateral to the arteriovenous fistula (Fig. 1).

Abdominal examination revealed left flank tenderness without hepatomegaly or splenomegaly. The rest of the examination was unremarkable.

The paraclinical workup, performed in the emergency room, showed pancytopenia, positive Coomb's test, normal reticulocyte count without signs of tumor infiltration on the bone marrow biopsy, an inflammatory syndrome with a CRP elevated to 80 mg/l, a blood smear and a brain scan without abnormalities. The echo-doppler ultrasound of the right upper limb was in favor of a partially thrombosed aneurysm of the right brachial artery.

To rule out an infectious origin, the biological workup requested, including lumbar puncture, urine cytobacteriological examination, chest radiograph, procalcitonin, blood culture, and COVID 19 PCR, was unremarkable.

**Fig. 2:**
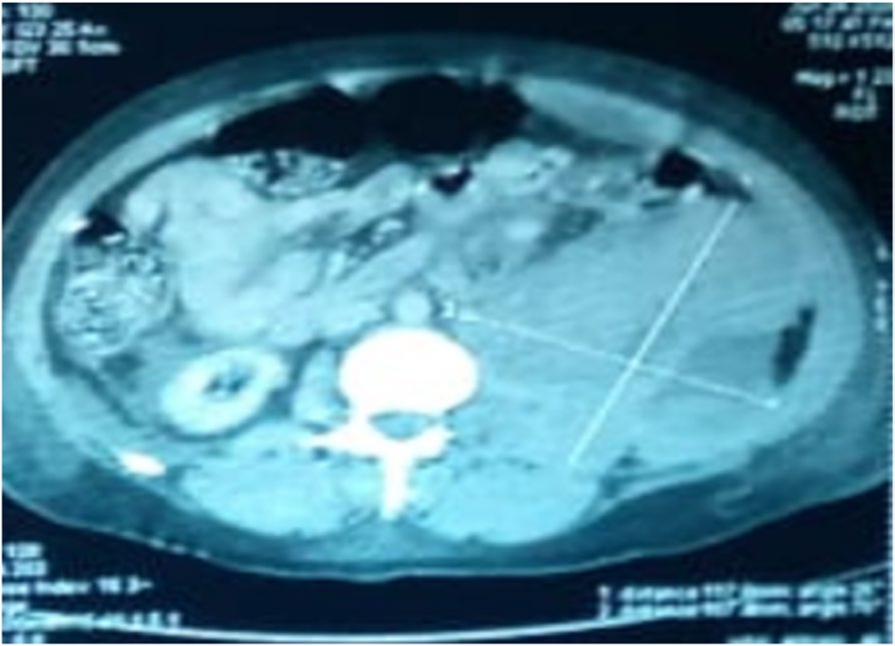
Abdominal-pelvic CT scan shows a large hematoma of the right psoas muscle

Abdominal ultrasound showed a huge collection of the left flank associated with other parietal collections in relation to hematomas. A supplemental abdominal-pelvic CT scan with contrast injection revealed a large psoas muscle hematoma measuring 220 mm × 180 mm, splenomegaly with circumferential pleuropericardial effusion (Fig. 2).

In view of the multisystemic involvement and thrombotic complications, an immunological assessment was requested, which showed a low level of complement C3 at 0.4 g/l [0.7 – 1.7 g], positive anti-native DNA antibodies, positive antinuclear antibodies and negative antiphospholipid antibodies.

The diagnosis of systemic lupus erythematous was retained in view of the hematological, cutaneous and renal involvement and a positive immunological assessment.

The decision of the multidisciplinary consultation staff was to diagnose a severe lupus attack on hemodialysis and to put the patient on a bolus of methylprednisolone, followed by oral treatment, with monthly tapering. The initial treatment was based on Mycophenolate mofetil (MMF) at a dose of 1 g/d.

The immediate evolution was favorable on the clinical and biological level with regression of the ecchymosis of the right arm and decrease of the inflammatory syndrome. Three months later, a follow-up abdominal ultrasound showed significant regression of abdominal hematomas with negativation of the immunological assessment at the 7th month of the attack, cessation of MMF and maintenance of a low dose of corticosteroid at 5 mg/day.

## Discussion

Systemic lupus erythematous (SLE) is a relatively common cause of the use of hemodialysis (HD) replacement therapy. It is well known that patients on chronic HD often have remission of clinical and serological features of the disease [1].

SLE activity decreases in patients with end-stage renal disease [2, 3]. Epidemiological studies of lupus extinction in dialysis are scarce, and none have described the consequences of lupus extinction on patient outcome. In dialysis, patients show lupus extinction at a median of 17 months [4].

However, the possibility of severe relapses during hemodialysis requires more regular monitoring and codified clinical, biological and immunological management [5].In our patient, the diagnosis of lupus was retained after two years of dialysis.

However, extra-renal symptoms related to disease activity are described, especially during the first year of dialysis [5]. Cutaneous, neurological, joint or hematological signs may be the first alarming signs of a lupus flare. Our patient presented with both clinical (pleuropericardial effusion, convulsions) and hematological manifestations.

One study showed that lymphopenia, factor C and S deficiency, presence of anti-cardiolipin antibodies, and discontinuation of anticoagulant therapy are the main predictive factors for lupus flare on dialysis [6].

Regarding the therapeutic management of lupus in hemodialysis, studies are rare and the modality varies according to the benefit/risk balance. Thus, lupus patients on chronic dialysis have a survival comparable to that of other dialysis patients [6].

## Conclusion

Through our observation, we can say that every patient in hemodialysis who presents multisystemic signs can hide a new push of his lupus disease, far from the hypothesis which supports the extinction of the disease after putting under hemodialysis. The possibility of lupus flares (20% to 50%), especially severe ones, in HD requires rigorous clinico-biological and immunological monitoring.

## Data Availability

The datasets used and/or analysed during the current study are available from the corresponding author on reasonable request.

## Abbreviations

CRP: C-reactive protein
SLE: Systemic lupus erythematous
MMF: Mycophenolate mofétil
